# P-2339. Unraveling the Genomic Diversity of Adenovirus Responsable for Severe Acute Respiratory Outbreak In Antioquia, Colombia, in 2022

**DOI:** 10.1093/ofid/ofae631.2491

**Published:** 2025-01-29

**Authors:** Maria Angelica Maya, Celeny Ortiz, Francisco Averhoff, Paulina Rebolledo, Ana Isabel Davila, Diego A Bastidas, Michael G Berg, Laura S Perez-Restrepo, Karl Ciuoderis, Jaime Usuga, Isabel Moreno López, Juan P Hernandez-Ortiz, Jorge Osorio

**Affiliations:** GHI One-Health Colombia, Universidad Nacional de Colombia, Medellín, Colombia, Medellin, Antioquia, Colombia; Gobernación de Antioquia, Medellin, Antioquia, Colombia; Abbott Laboratories, Atlanta, Georgia; Emory University School of Medicine, Emory University Rollins School of Public Health, Atlanta, GA; San Vicente Fundacion Hospital, Medellin, Antioquia, Colombia; Hospital Universitario San Vicente Fundación, Medellín, Antioquia, Colombia; Abbott Labs, Abbott Park, Illinois; GHI One-Health Colombia, Universidad Nacional de Colombia, Medellín, Colombia, Medellin, Antioquia, Colombia; Universidad Nacional de Colombia, medellin, Antioquia, Colombia; GHI One Health Colombia, Universidad Nacional de Colombia, Medellin, Colombia, Medellin, Antioquia, Colombia; Universidad Nacional de Colombia, medellin, Antioquia, Colombia; Universidad Nacional de Colombia, medellin, Antioquia, Colombia; Global Health Institute, Madison, Wisconsin

## Abstract

**Background:**

Adenovirus (HAdV) is a common cause of acute respiratory infection (ARI). In healthy people, infections are usually mild and self-limiting. However, during the second half of 2022, an HAdV severe acute respiratory infection (SARI) outbreak occurred in Colombia. Although HAdV epidemiological data in South America is scarce, SARS-CoV-2 pre-pandemic studies reported HAdV-B3 as the main cause of respiratory adenoviral infections. Our study investigated the circulating HAdV genotype before, during, and after this outbreak along with clinical data associated.**Figure 1.** Tracking of HAdV Severe Acute Respiratory Infection in Antioquia, February 2022 to April 2023

**Figure:**
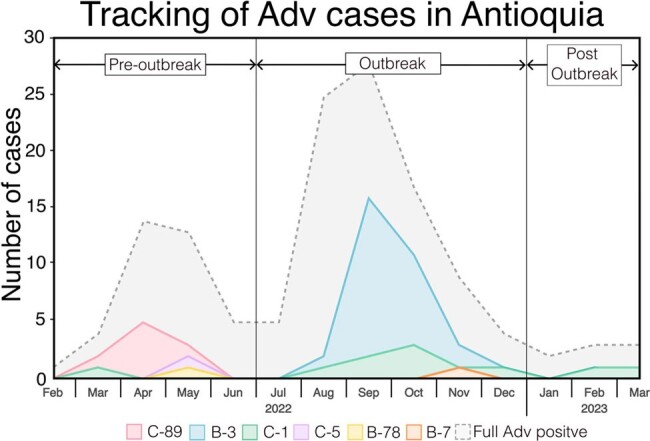

The quarterly distribution of various human adenovirus genotypes in Antioquia-Colombia starting from the first quarter of 2022 to the second quarter of 2023. Areas represent the percentage prevalence of strains obtained in each quarter: HAdV-C89 (red), HAdV-C1 (green), HAdV-B78 (yellow), HAdV-C5 (purple), HAdV-B3 (blue), HAdV-B7 (orange), and all HAdV SARI cases enrolled (grey).

**Methods:**

During February 2022 and April 2023, children with HAdV SARI were enrolled after medical attention. Inclusion criteria were respiratory symptoms onset within 15 days and HAdV PCR positive test result. After written informed consent, a nasopharyngeal aspirate was collected. Respiratory Pathogen ID/AMR Enrichment Panel Kit was used to sequence samples collected before the outbreak while those collected during and after the outbreak were sequenced using hexon gene-specific primers. Descriptive analysis of study data and phylogenetic analysis of the sequences were done.

**Table 1.**
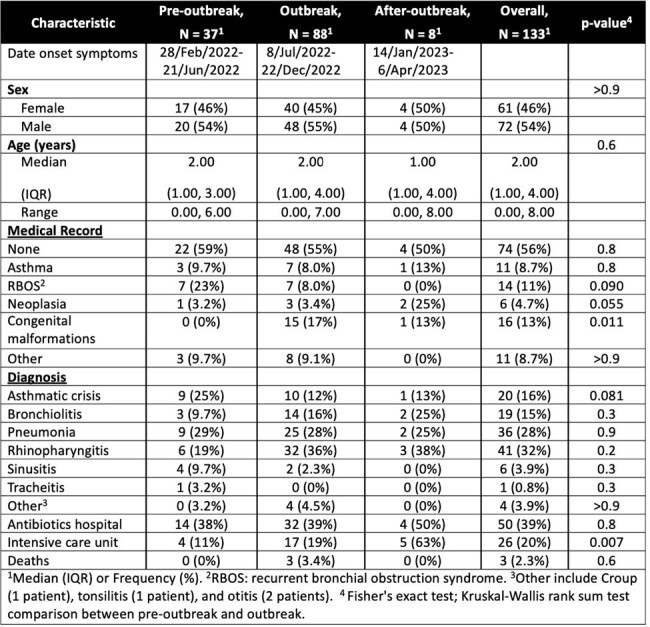
Clinical Characteristics of Children with HAdV Acute Respiratory Infection in Antioquia in the Pre-Outbreak, Outbreak and After-Outbreak

**Results:**

A total of 133 patients were enrolled, from whom 54 samples were sequenced. The outbreak started in the second week of July, reached its peak between August and September, and concluded by the end of December. The median age was 2 years old (IQR 1-4). During the outbreak, 63/88 patients had comorbidities, 25/88 developed pneumonia, and 17/88 required critical care attention. Pre-outbreak period, most sequences (71.4%, 10/14) were phylogenetically classified as the novel genotype HAdV-C89, while during and after the outbreak the majority (85.0%, 34/40) were HAdV-B3 genotype.**Figure 2.** Serotype and Genotype Phylogenetic Relationships of HAdV

**Figure:**
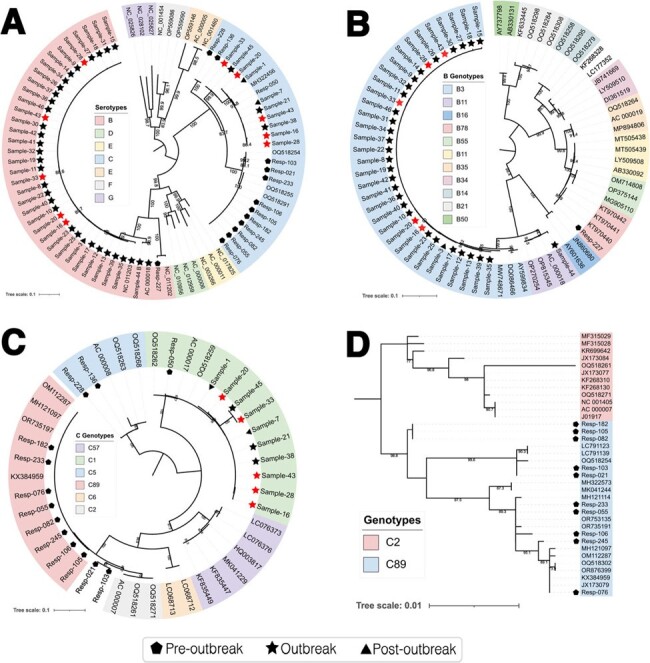

Maximum Likelihood trees were constructed using the IQ-Tree V 2.3.3 program with ultrafast bootstrapping (UFBoot) and 1,000 replicates. ModelFinder was calculated as the best-fit substitutional model according to Bayesian information criteria. Strains from this study were categorized by symbols corresponding to their respective batches: pentagons, stars, and circles representing strains from pre-outbreak, outbreak, and post-outbreak respectively. Red stars correspond to co-detection B3/C1. Each tree was constructed as follows: A) Phylogenetic tree of partial hexon sequence from A – G HAdV serotypes. B) B Genotypes phylogenetic tree of hexon partial sequence. C) C Genotypes phylogenetic tree of hexon partial sequence. D) C2 and C89 Genotypes phylogenetic tree of full penton sequence. Bootstrap values greater than 80 are shown in the branches.

**Conclusion:**

Our result indicated that the novel HAdV-C89 genotype was causing SARI in Antioquia (Colombia) during the first half of 2022, after it was displaced by the re-emergence of HAdV-B3, causing an increase of HAdV SARI. Once discontinuing the pandemic measures, commonly known respiratory pathogens can re-emerge, therefore monitoring of those should be intensified.

**Table 2.**
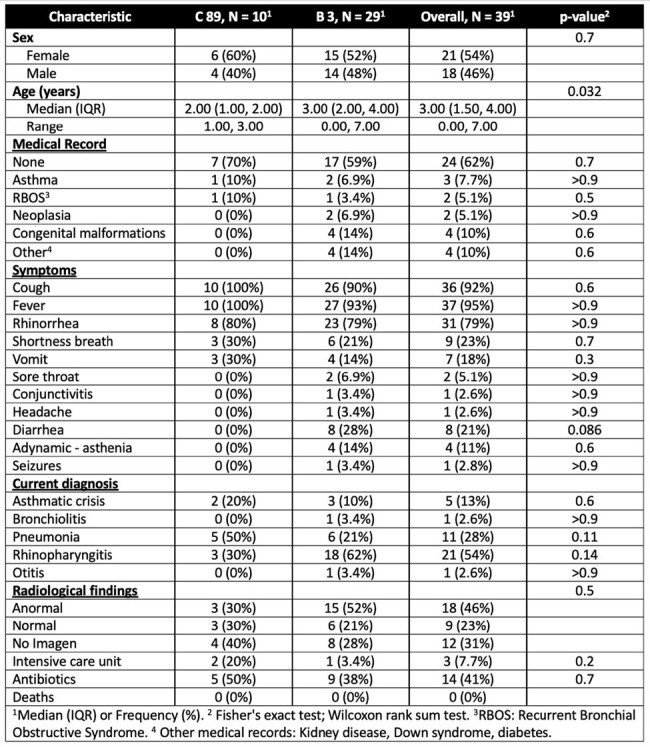
Comparison of Demographic, Clinical Features, and Outcomes between Children with HAdV-C89 and B3 Respiratory Infection

**Disclosures:**

Maria Angelica Maya, MD, Abbott: Grant/Research Support Francisco Averhoff, MD, MPH, Abbott Laboratories: Employee Michael G. Berg, PhD, Abbott Laboratories: employee|Abbott Laboratories: Stocks/Bonds (Public Company)

